# Preimplantation genetic diagnosis for TTR-FAP in Portugal

**DOI:** 10.1186/1750-1172-10-S1-I5

**Published:** 2015-11-02

**Authors:** Filipa Carvalho

**Affiliations:** 1 Department of Genetics, Faculty of Medicine, University of Porto, Portugal; 2 Instituto de Investigação e Inovação em Saúde, Universidade do Porto, Portugal

## Background

Portuguese Familial Amyloidotic Polyneurophaty (FAP) (OMIM #105210) is a rare systemic amyloidosis characterized by a progressive, autonomic and sensory-motor neuropathy. FAP is an autosomal dominant disorder caused by mutations in the transthyretin (*TTR*) gene. Preimplantation genetic diagnosis (PGD) is currently one of the options available for couples at-risk to avoid disease transmission. The review the PGD cycles for TTR-FAP performed in the last 15 years in Portugal will be presented.

## Methods

Embryos were biopsed at day 3 of development and diagnosis was initially performed using fluorescent PCR primers designed to amplify the p.Val50Met in exon 2 of the *TTR* gene and is currently done by multiplex PCR for the mutation detection and polymorphic markers.

## Results

Two hundred and thirty-five clinical cycles were performed in 118 couples (74 with paternal and 44 with maternal mutation), with a mean number of 2 cycles per couple and a mean maternal age of 31.4 years old. The mean number of MII injected oocytes per cycle was 8.7. Fertilization and cleavage rates were 74.5% and 98.2%, respectively. Eighty-nine percent of embryos were biopsied (mean number = 5.8) and amplification was obtained in 94% with a ratio of normal *versus* mutated embryos of 1:1,3 (p < 0.0001). Interestingly, if only the cycles of paternal transmission were considered the ratio of normal *versus* mutated embryos was 1:1,03 (p=0,7018) whereas if the cycles of maternal transmission were analyzed the ratio of normal *versus* mutated embryos was 1:1,77 (p<0,0001). One hundred and fifty-eight cycles had embryo transfer (mean embryo number = 1.7) leading to 57 biochemical (36%) and 50 clinical pregnancies (32%) (45 term pregnancies, 3 ongoing pregnancies and 2 miscarriages). Three pregnancies correspond to frozen embryos transfers. Thirty-seven term pregnancies were singleton (mean gestational age = 38.4W; average weight at birth = 3093g), 7 were twins (mean gestational age = 34.5W; average weight at birth = 2310g) and one was a triplet pregnancy (two embryos transfer) (gestational age = 34W; average weight at birth = 1700g).

## Conclusion

PGD for Portuguese type TTR-FAP is a well established procedure allowing the birth of unaffected children with a take-home baby rate similar to the one described in the literature.

